# Profits First, Health Second: The Pharmaceutical Industry and the Global South: Comment on "More Pain, More Gain! The Delivery of COVID-19 Vaccines and the Pharmaceutical Industry’s Role in Widening the Access Gap"

**DOI:** 10.34172/ijhpm.2024.8471

**Published:** 2024-05-18

**Authors:** Joel Lexchin

**Affiliations:** ^1^School of Health Policy and Management, York University, Toronto, ON, Canada.; ^2^Department of Family and Community Medicine, University of Toronto, Toronto, ON, Canada.

**Keywords:** Access, Pandemic Accord, Pharmaceutical Industry, Research and Development

## Abstract

The pharmaceutical industry has a long history of prioritizing the research and sale of medicines that will yield the largest amount of revenue and placing the health of people second. This gap is especially prevalent in countries of the Global South. This article first explores the dichotomy in research between the Global North and the Global South and then looks at examples of how access to key medicines used in diseases such as HIV, oncology and hepatitis C is limited in the latter group of countries. The role of pharmaceutical companies during the COVID-19 pandemic prompted negotiations for a pandemic accord that would ensure more equity in both research and access when the next pandemic comes. However, efforts by a combination of the pharmaceutical industry and some high-income countries (HICs) are creating serious obstacles to achieving the goal of an accord that would place health over profits.

## Introduction

 The article by Borges and colleagues^1^ about the inequitable access to COVID-19 vaccines between the Global North and the Global South is a stark reminder that pharmaceutical companies are primarily motivated by profits with the alleviation of suffering and the promotion of health a secondary concern. Borges et al focus on access, but the profit motivation also determines the choice of conditions that companies undertake research and development (R&D) for. Access is meaningless unless R&D has produced drugs worth accessing.

 This commentary first addresses the biases in R&D and then pivots to examine access to HIV, oncology and hepatitis C medicines. Finally, it picks up from where Borges et al left off to document the state of the negotiations over the World Health Organization (WHO) Pandemic Accord that is supposed to help remedy inequitable access to vaccines, treatments, diagnostics and technology. The underlying theme throughout this commentary is that the current system, based on maximizing profitability, does not serve the interests of the people who live in the Global South.

## Research and Development

###  Drugs for Neglected Diseases Versus Drugs for Orphan Diseases

 There are an estimated 1 billion people annually who suffer from neglected tropical diseases. In contrast, there are between 263 to 446 million people affected by an orphan disease. But in 2021, pharmaceutical companies contributed US$ 580 million out of a total of $4.137 billion spent on R&D for neglected diseases^2^ or $0.58 per capita versus spending $35.3 billion or $100 per capita on R&D for orphan diseases.^3,4^

 The near abandonment of R&D for drugs to treat neglected diseases is the main reason why between 2000 to 2011 of the 850 new therapeutic products registered internationally, just 37 (4%) were indicated for neglected diseases and only four new chemical entities were approved for neglected diseases.^5^ The situation has improved somewhat since 2011. The 20 largest pharmaceutical companies are now collectively developing more than twice as many medicines needed by people living in low- and middle-income countries compared to 2014. However, five of those companies accounted for 63% of the most urgently needed R&D projects and the R&D was only focused on a small number of high-burden and/or high-priority diseases.^6^

###  Clinical Trials and Registration

 Even when clinical trials of new drugs are conducted in countries in the Global South, companies frequently do not apply to register the drugs in those countries. Out of 33 drugs that had test sites in Latin America and were subsequently approved by the United States Food and Drug Administration (FDA), just 8 were registered and commercialized in all the Latin American countries where they had been tested and 10 had not been registered in any of the countries.^7^ In a second study that examined countries where testing of 34 drugs took place, at 5 years post FDA approval access rates were 9% (2 of 22) in upper-middle income countries) and 22% (2 of 9) in lower-middle income countries.^8^

###  COVID-19 Versus Ebola

 The spectacular success of pharmaceutical companies in rapidly developing multiple COVID-19 vaccines, albeit with billions of dollars in public research funding, contrasts markedly with their approach to a vaccine for Ebola, an infectious disease largely confined to West Africa. The initial development of a vaccine was done in a Canadian government laboratory in the early 2000s until it was licensed to a small US biotechnology company where it essentially sat without any further work being done. It was only the outbreak of 2014-2016 that raised fears that Ebola might spread outside of Africa to high-income countries (HICs) that accelerated activity to bring the vaccine to market.^9^

###  Pharmaceutical Company Chief Executive Officers on R&D

 The reality of how profits and R&D are intertwined is revealed in quotes from two pharmaceutical company chief executive officers (CEO). The first, Daniel Vasella, when he was CEO of Novartis, acknowledged that “*You can’t expect for-profit organisation to do this [produce new drugs for developing countries]...If you want to establish a system where companies systematically invest in this kind of area, you need a different system.*”^10^ The second and much more dramatic quote is from Marijn Dekkers, CEO of Bayer, commenting on the compulsory license issued by India for his company’s oncology drug Nexavar (sorafenib): “*We did not develop this product for the Indian market, let’s be honest...I mean, you know, we developed this product for Western patients who can afford this product, quite honestly.*”^11^

## Access to Vaccines and Medicines

###  HIV, Oncology, and Hepatitis C Medicines

 Borges et al comprehensively document the inequitable access to COVID-19 vaccines but the issue of unequal availability of vaccines and medicines goes back many decades and came prominently to global attention in the late 1990s. At that time, triple therapy for HIV/AIDS cost about US$ 10 000 per person per year making it virtually unaffordable to the vast majority of the population in countries of the Global South where the prevalence of infection was the greatest.

 Faced with increasing rates of HIV infection and the high prices for HIV treatment, the South African government passed the Medicines and Related Substances Control Amendment Act that allowed for generic substitution of off-patent medicines, transparent pricing for all medicines, and the parallel importation of patented medicines. In response, 39 multinational pharmaceutical companies, with the support of the US government and the European Commission, took the South African government to court in 1998. Eventually, in the face of widespread public opposition, the US government withdrew its support for the court case and without the US support the companies dropped their lawsuit.

 In early 2007, following failed negotiations with Abbott over the price of its combination antiretroviral drug Kaletra (lopinavir/ritonavir), the Thai Ministry of Public Health started the process of issuing a compulsory license for the drug. In response, Abbott announced that it would stop registering all its new medicines in Thailand, stating that “Thailand has chosen to break patents on numerous medicines, ignoring the patent system. As such, we’ve elected not to introduce new medicines there.”^12^

 Far more people have access to medicines for HIV now than they did in the earlier part of the century largely due to the availability of generics, but access is frequently just for first-line therapies. Second- and third-line drugs are often still protected by patents limiting availability.^13^

 Novartis’s Glivec/Gleevec (imatinib) is a major advance in the treatment of chronic myeloid leukemia but is priced at a level that would exclude the majority of people in India with the condition from being able to afford it. In contrast, an Indian made generic version costs 1/25 as much as Novartis’s brand-name version. Novartis attempted to block the generic from being sold by patenting Glivec, but after a 7-year battle, the Indian Supreme Court ultimately ruled against Novartis. Novartis’ reaction was that the “decision … discourages innovative drug discovery essential to advancing medical science for patients” and the company “will be cautious in investing in India especially with regard to introduction of innovative medicines.”^14^

###  Tiered Pricing and Generic Licensing

 A recent initiative by the large pharmaceutical companies to stave off compulsory licensing and to regain public trust in their commitment to accessibility has been tiered pricing, whereby companies offer their products at different prices depending on a country’s gross domestic product per capita. However, middle-income countries such as India and Brazil are often excluded from tiered pricing schemes despite substantial portions of their populations living below the poverty line. Moreover, in many cases, competition has meant that generic prices are lower than the tiered prices being offered. Gilead cut the price of its breakthrough hepatitis C drug Sovaldi (sofosbuvir) from US$ 84 000 per course of treatment in the United States to US$ 900 in Egypt, India, and Pakistan. However, Sovaldi is inexpensive to manufacture and could be sold at a profit by generic companies for US$ 150-300.

 In the mid-2010s, pharmaceutical companies instituted 53 price reduction strategies in an effort to improve access to medicines that they make in the Global South, but despite claims by companies of a positive impact, 94% of the evaluations of these projects were of low or very low quality and as a result whether these interventions were successful is unknown.^15^

 Moon et al summarized the problems with tiered pricing: “First, tiered pricing does not necessarily result in the lowest sustainable prices…Second, no clear international norm has been established for setting price tiers…Finally, tiered pricing policies give too little decision-making power to governments, which are accountable to their populations under international law for ensuring access to medicines.”^16^

 Similar problems exist when brand-name companies issue voluntary licenses for their medicines to generic companies located in the Global South. While the availability of generics is welcome, these licenses come with restrictions on where the resulting generics can be sold. In a move to head off a possible rejection of its patent in India that would allow unrestricted generic competition, Gilead issued licenses to 11 companies there to sell Sovaldi in 91 countries, but excluded from that list were some middle-income countries like Argentina, Brazil, China, Russia, and Ukraine.

## World Health Organization Pandemic Accord

 The failure to share research especially about COVID-19 vaccines and to ensure equitable access to them, as robustly shown by Borges et al, led to a special session of the World Health Assembly in late November 2021. At that session, it was agreed to establish an Intergovernmental Negotiating Body to draft and negotiate a WHO agreement on pandemic prevention, preparedness and response that would foster an all of government and all of society approach to future pandemics. Provisions in the treaty would include greatly enhancing international co-operation to improve, for example, alert systems, data-sharing, research and local, regional and global production and distribution of medical and public health counter-measures such as vaccines, medicines, diagnostics and personal protective equipment.

 Negotiations for the treaty are supposed to be finalized by May 2024, but even if that unlikely deadline is reached, the resulting treaty may be a far cry from fulfilling its ambitious goals. During the negotiations on the Zero-Draft version, 24 pharmaceutical company CEOs from the Biopharmaceutical Roundtable criticized the inclusion of intellectual property (IP) rights waivers and pathogen benefit sharing. They argued that the treaty as it stood would make the world less prepared for the next pandemic by threatening IP rights and slowing the pace of pathogen sequence sharing. According to the president of the International Federation of Pharmaceutical Manufacturers and Associations (IFPMA) “[Protection of] intellectual property rights (IP) I think is one of the lessons from COVID-19.” The CEO of Eli Lilly maintained that “IP was never an issue for access in low and middle income countries” and that “We must prevent the weakening of the international IP protections that would result from unnecessary and misguided proposals to waive the TRIPS agreement for vaccines and therapeutics.” The industry CEOs also objected to the provision in the Zero-Draft that would allow countries, often those in the Global South, that shared genetic sequences to seek financial compensation for uploading them to open databases. The position of the IFPMA Director General was that “Such approaches are more than likely to delay access to pathogens and the timely development of medical countermeasures in the event of a pandemic.”^17^

 The position of the pharmaceutical companies is being reinforced by some HICs. There are European countries that are arguing that the WHO is not the right forum for discussions about IP rights and that these talks should be taking place at the World Trade Organization. Other HICs are objecting to the inclusion of provisions about openly sharing the results of research and about affordable pricing in contracts between public funders and researchers. The position of the HICs is that it could be complicated to include such conditions in research-funding contracts.^18^

 The next draft of the treaty (the Negotiating Draft) reflected the influence of the opponents to a treaty that would support equitable sharing and access. As one example, the Zero-Draft proposed some conditions, favourable to Global South countries, to be included in research-funding contracts, including on prices of products, data sharing and the transfer of technology during a pandemic. The Negotiating Draft omitted these provisions and instead merely said that governments should “publish the terms of government-funded research and development agreements for pandemic-related products” but did not specify what the terms should be.^18^ The Negotiating Draft also used weak language about accessing undisclosed information stemming from research; instead of using strong language such as “shall” or “must” in many clauses, it used more permissive terms like “encourage” or “should.”

## Conclusion

 Pharmaceutical companies have a long history of prioritizing the financial interests of their shareholders above the health of the people who use their products, and this is especially true for those living in the Global South. Companies do not engage in R&D for diseases that are largely prevalent in Global South countries and make little attempt to ensure that their products are available and affordable there. The poverty of access to vaccines for COVID-19 in Global South countries is just the latest manifestation of the paradigm driving the pharmaceutical industry. The prospect of a WHO-backed pandemic accord has the potential to change that paradigm, but it will require worldwide mobilization by those who value health equity to achieve that end.

## Ethical issues

 Not applicable.

## Competing interests

 Between 2020-2024, Joel Lexchin received payments for writing a brief on the role of promotion in generating prescriptions for a legal firm, for being on a panel about pharmacare and for co-writing an article for a peer-reviewed medical journal. He is a member of the Board of Canadian Doctors for Medicare. He receives royalties from University of Toronto Press and James Lorimer & Co. Ltd. for books he has written. He is participating in research funded by the Canadian Institutes of Health Research.
